# Microscopic insight into the origin of super-cooled NCCDW state in 1T-TaS₂ nanocrystals

**DOI:** 10.1038/s41598-026-42525-9

**Published:** 2026-03-25

**Authors:** Georgios Chatzigiannakis, Anastasia Soultati, Elias Sakellis, George Papageorgiou, Nikos Boukos, Vassilis Psycharis, Catherine P. Raptopoulou, Konstantinos Aidinis, Spiros Gardelis, Alexander Chroneos, Maria Vasilopoulou

**Affiliations:** 1 Institute of Nanoscience and Nanotechnology, National Center for Scientific Research “Demokritos”, Agia Paraskevi, 15341 Athens, Greece; 2 Section of Condensed Matter Physics, Department of Physics, National and Kapodistrian University of Athens, Panepistimioupolis, Zografos, 15784 Athens, Greece; 3 Department of Electrical and Computer Engineering, Ajman University, P.O. Box 346, Ajman, United Arab Emirates; 4 Center of Medical and Bio-Allied Health Sciences Research, Ajman, United Arab Emirates; 5 Department of Electrical and Computer Engineering, University of Thessaly, 38221 Volos, Greece; 6 Department of Materials, Imperial College, London, SW7 2AZ UK

**Keywords:** 1T-TaS_2_, Phase transitions, Charge density waves (CDW), Metastable phases, Super-cooled NCCDW phase, Structural characterization, Materials science, Nanoscience and technology, Physics

## Abstract

Tantalum disulfide (1T-TaS₂) is a quasi-two-dimensional transition metal dichalcogenide (TMD) that exhibits a series of charge density wave (CDW) transitions upon cooling and heating. These collective electronic phases can be tuned or disrupted by external stimuli such as pressure, electric fields, or illumination, leading to metastable or hidden metallic states. In nanoscale crystals of 1T-TaS₂, rapid cooling suppresses the insulating commensurate CDW (CCDW) phase and stabilizes a metastable metallic state, known as the super-cooled nearly commensurate CDW (SC-NCCDW) phase. However, the atomic-scale structure and microscopic origin of this state remain elusive. Here, we combine electrical transport measurements with structural characterization to elucidate the nature of the SC-NCCDW phase in 1T-TaS₂ nanocrystals. Temperature-dependent X-ray diffraction reveals that, under gradual cooling, the NCCDW-to-CCDW transition is accompanied by lattice-volume expansion. In contrast, this anomaly is strongly suppressed upon rapid cooling, correlating with the stabilization of the SC-NCCDW state. Complementary high-resolution transmission electron microscopy (HR-TEM) shows that rapid cooling produces a mixed-phase configuration containing structural motifs of both NCCDW and CCDW phases, indicating that the SC-NCCDW represents an intermediate structural configuration frozen by kinetic constraints. These findings provide, to our knowledge, the first direct structural evidence of the SC-NCCDW state and offer a mechanistic understanding of cooling-rate-controlled metastability in layered correlated compounds such as 1T-TaS₂.

**Supplementary Information:**

The online version contains supplementary material available at 10.1038/s41598-026-42525-9.

## Introduction

Transition metal dichalcogenides (TMDs) have attracted great interest in condensed matter physics and material science due to their unique mechanical, optical and electrical properties derived from their layered quasi-two-dimensional (2D) crystal structure^1^. Among these materials, the 1 T polymorph of tantalum disulfide (1T-TaS₂) stands out for exhibiting a rich sequence of charge-density-wave (CDW) states that evolve with temperature and external perturbations^2–5^. CDWs are collective electronic states arising from the interplay between electron correlation, lattice strain, and Fermi surface nesting, and they are also observed in other layered TMDs like 2 H-TaS_2_, 1T-TaSe_2_, 2 H-TaSe_2_, 1T-NbS_2_, 1T-ReS_2_ and so on^3,6–17^. The tunability of these states renders TMDs, and especially 1T-TaS₂, ideal systems for investigating emergent electronic phases and their potential use in nanoelectronic applications.

In 1T-TaS₂, successive temperature-driven CDW transitions are intricately coupled to lattice distortions that form star-shaped polaron clusters known as ‘’David stars’’ in which twelve Ta atoms contract towards a thirteenth central Ta atom^16,17^. At high temperatures (~ 550 K), the material is metallic and lacks such clustering while on cooling to ~ 350 K, it enters the nearly commensurate CDW (NCCDW) phase containing ordered David-star clusters separated by metallic domain walls. Further cooling below ~ 180 K yields the fully commensurate CDW (CCDW) phase with a √13×√13 superlattice of David-star units. The accompanying periodic lattice distortion opens an energy gap and causes a strong increase in resistivity. The low-temperature insulating state was originally interpreted as a Mott–Hubbard insulator arising from electron localization within the David-star clusters^2,17–21^. More recent studies, however, have revealed that interlayer stacking arrangements, orbital textures, and band-structure effects also play decisive roles in gap formation^22–25^. Overall, the insulating ground state of the CCDW phase is now considered a cooperative outcome of structural distortions, interlayer stacking, and electron–electron interactions, instead of a purely Mott-related gap.

These CDW phases are highly sensitive to external perturbations such as optical pulses, electrical pulsing, gating, pressure, irradiation, or strain^26–36^. Such stimuli can tune, melt, or suppress long-range CDW order, giving rise to hidden or metastable metallic states that are absent from the equilibrium phase diagram. Ultrafast optical excitation^37,38^, strong electric fields^39–42^ or electrical pulsing^29,39^, gating^43,44^, and pressurization^27,45,46^ have all been shown to drive the system into non-equilibrium metallic states at low temperatures. The pronounced tunability of these states makes 1T-TaS₂ an exceptional platform for exploring non-equilibrium phase transitions and metastability in correlated electron systems. Beyond optical and pressure-driven control, device-oriented studies have shown that electrical bias, Joule heating and electrostatic gating can also influence CDW dynamics in thin 1T-TaS₂ films, particularly affecting domain depinning and the NC–IC switching window^47–49^. These works further highlight the kinetic nature of CDW transitions in reduced dimensions, complementing our focus on cooling-rate-controlled stabilization of the low-temperature NCCDW→CCDW transition.

In exfoliated nanothick crystals, the kinetics of ordering become particularly important. Yoshida et al.^44^, demonstrated that rapid cooling of thin 1T-TaS₂ crystals suppresses the insulating CCDW and traps the system in a metastable low-resistance configuration—the super-cooled nearly commensurate CDW (SC-NCCDW) phase—whereas bulk crystals exhibit the usual sequence of transitions independent of cooling rate. This behavior underscores the role of reduced dimensionality and altered stacking in governing the kinetics of CDW ordering and the emergence of non-equilibrium phases. In addition to these thickness-dependent effects, Riffle et al.^50^, recently examined the influence of cooling rate in bulk 1T-TaS₂ single crystals using low-temperature scanning tunneling microscopy (STM) and x-ray diffraction (XRD), comparing slow (< 1 K min⁻¹) and very fast (> 50 K min⁻¹) continuous cooling from room temperature. They found that, in almost all cases, the low-temperature state remained the conventional CCDW phase, independent of cooling rate, with only rare fast-cooled samples exhibiting metallic mosaic or multi-chiral CDW configurations. These results indicate that, in bulk crystals, cooling rate alone does not provide a reliable route to bypass the equilibrium CCDW ground state.

While many aspects of 1T-TaS₂ phase transitions are well studied, the structural changes induced by rapid cooling—and thus the microscopic origin of the super-cooled NCCDW state, especially in exfoliated nanocrystals—remain unclear. Here we address this question by combining electrical transport measurements with temperature-dependent single-crystal XRD and high-resolution transmission electron microscopy (HR-TEM) on 1T-TaS₂ nanocrystals, with thick flakes included as a bulk-like reference. Electrical measurements confirmed the well-known sequence of CDW transitions under gradual cooling and the suppression of insulating CCDW state under rapid cooling in thin flakes while bulk crystals remained unaffected by cooling rate, in agreement with the previous report of the metallic SC-NCCDW phase^44^. Single-crystal XRD analysis of thin flakes revealed a distinct expansion of the unit-cell volume near 180 K during gradual cooling, whereas this anomaly was strongly suppressed following rapid cooling, correlating with the stabilization of the SC-NCCDW state. HR-TEM images of fast-cooled crystals and their corresponding fast Fourier transforms (FFTs) revealed the co-existence of regions demonstrating diffraction spots characteristic of CCDW and larger regions exhibiting diffraction spots typical of the NCCDW phase similar to the original sample. This mixed pattern indicates that the SC-NCCDW phase is actually an intermediate phase consisting of CCDW nanodomains embedded within the NCCDW matrix. Together these results provide, to the best of our knowledge, the first direct structural insight into the SC-NCCDW phase in 1T-TaS₂ nanocrystals, thereby advancing the understanding of cooling-rate–controlled metastability in this prototypical CDW material.

## Results

### Exfoliation of 1T-TaS_2_ nanocrystals: structure and morphology

**Fig. 1:**
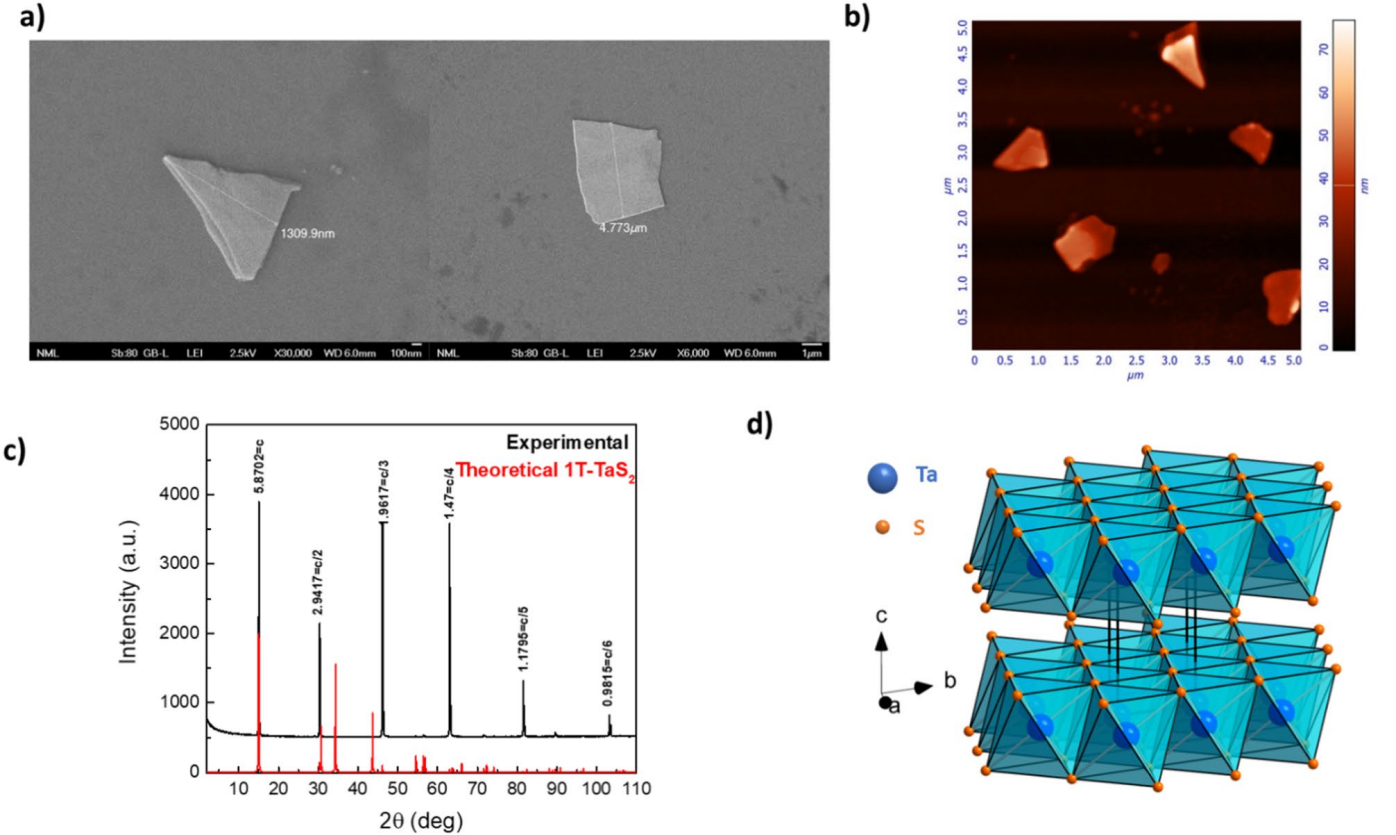
Structural and morphological investigation of exfoliated 1T-TaS_2_ nanocrystals. **a** SEM micrographs of two representative exfoliated 1T-TaS_2_ crystals with lateral size 1–5 μm. **b** AFM micrograph of exfoliated 1T-TaS_2_ crystals of 10–70 nm thickness. **c** Powder XRD pattern of exfoliated 1T-TaS_2_ crystals (black line) and the simulated pattern (red line) derived from the crystallographic data of 1T-TaS₂ (ICSD No. 651089, P −3 m 1). The experimental pattern confirms 1 T polytype and the c-axis layered stacking. **d** Schematic representation of the 1T-TaS₂ layered structure, where Ta atoms (blue) are sandwiched between S atoms (orange) forming edge-sharing octahedra.

To elucidate the structural origin of the cooling-rate–dependent electronic behavior observed in 1T-TaS₂ nanocrystals we used Scotch-tape method to mechanically exfoliate thin flakes from a bulk-commercial single-crystal. The morphology of the exfoliated crystals was investigated by scanning electron microscopy (SEM) and atomic force microscopy (AFM) (Fig. 1a and b) revealing well-defined flake-like crystals with lateral sizes of 1–5 μm and thicknesses from a few nanometers to several tens of nanometers.

The crystal structure of the exfoliated 1T-TaS₂ nanocrystals was examined by powder X-ray diffraction (XRD). The XRD pattern (Fig. 1c) exhibits sharp (00 L) reflections corresponding exclusively to the 1 T phase, confirming the high crystallinity, structural purity, and preferred orientation of the layers along the c-axis. The absence of in-plane diffraction peaks indicates that the broad surfaces of the flakes are parallel to the ab-plane, consistent with strong texturing typical of layered dichalcogenides. The atomic structure of 1T-TaS₂, illustrated in Fig. 1d, consists of Ta atoms octahedrally coordinated by S atoms. These octahedra share edges to form layers extending parallel to the (001) plane, which are stacked along the crystallographic c-axis, giving rise to a quasi-two-dimensional structure. The preservation of 1 T polytype after the mechanical exfoliation was also confirmed by room-temperature Raman investigation exhibiting the characteristic modes of the material^51^ (Supplementary Information, Fig. S1).

Together, these morphological and structural characterizations demonstrate that the exfoliated flakes preserve the intrinsic layered structure of 1T-TaS₂, providing a reliable platform for investigating their temperature- and cooling-rate–dependent electronic and structural behavior.

### Electrical investigation of 1T-TaS_2_ nanocrystals

**Fig. 2:**
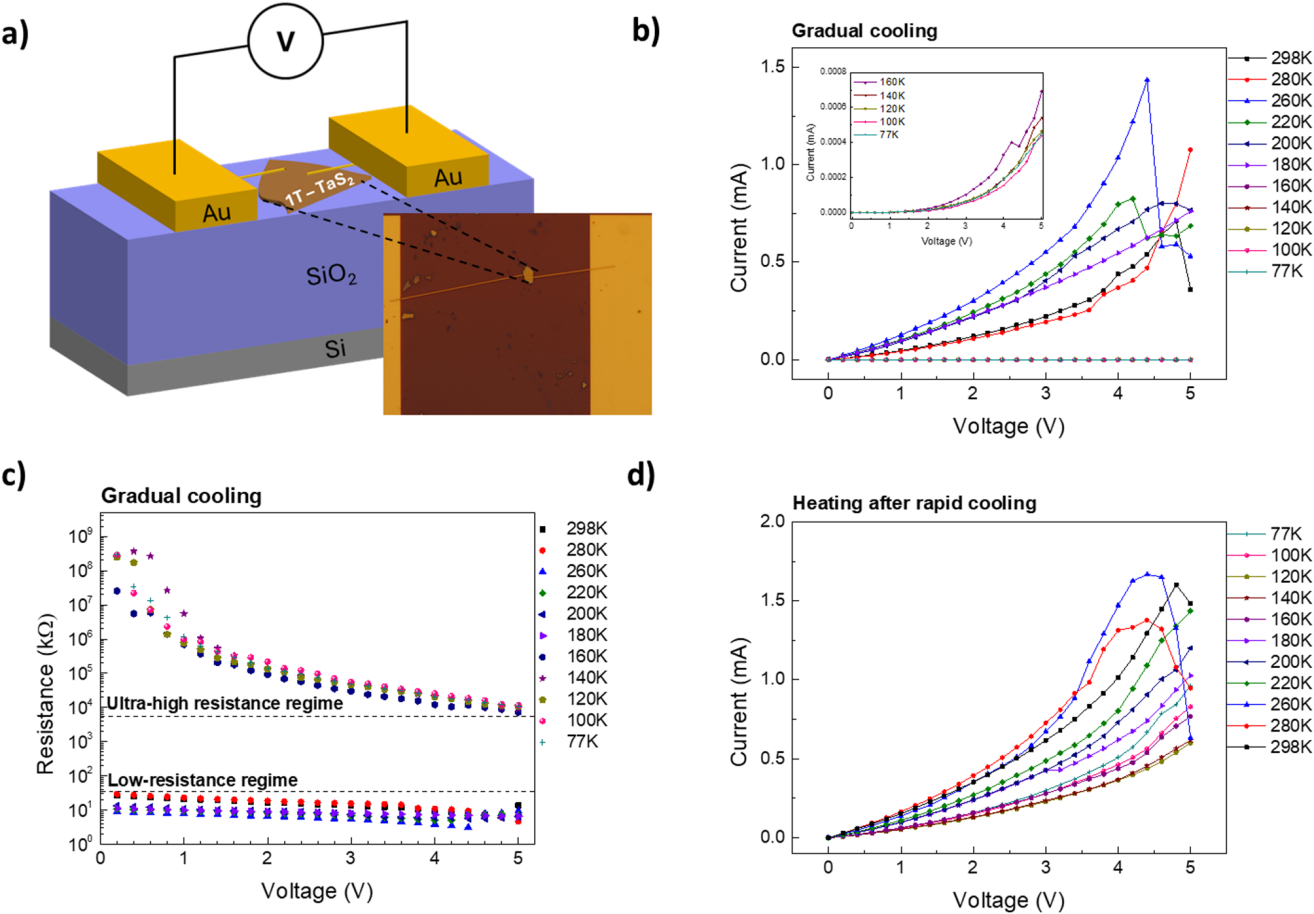
Temperature-dependent electrical characterization of a 1T-TaS₂ nanocrystal device. **a** Schematic illustration of the two-probe device configuration with Au electrodes on a Si/SiO₂ substrate. The inset shows an optical micrograph of the fabricated 1T-TaS₂ nanocrystal device. **b** Current–voltage (*I–V*) characteristics measured at various temperatures during gradual cooling. The inset highlights the low-current regime. **c** Resistance–voltage (*R–V*) characteristics extracted from the *I–V* data in panel (b). **d** *I–V* characteristics measured at various temperatures during gradual heating following rapid cooling to 77 K.

To explore the temperature-dependent transport properties of the exfoliated 1T-TaS_2_ nanocrystals two-probe current–voltage (*I–V*) measurements were performed. The devices were fabricated by transferring flakes onto Si/SiO₂ substrates and patterning Au contacts via electron-beam lithography and metal evaporation (see Methods and Supplementary Information, Fig. S2). A schematic and an optical micrograph of a representative device are shown in Fig. 2a. Details of the fabrication process and measurement configuration are provided Methods.

Figures 2c-d summarize the electrical transport properties of a representative 1T-TaS₂ nanocrystal, highlighting the influence of cooling rate on CDW transitions. Figure 2b shows the *I-V* characteristics during gradual cooling from 298 K to 77 K at an average cooling rate of 1 K min^− 1^. At high temperatures, the device exhibits a nearly ohmic (linear) behavior in the low-voltage region, consistent with the metal-like character of 1T-TaS_2_ crystals in this range. Upon cooling below 180 K, a sharp drop in current is observed, approaching nearly zero, indicating a transition to a highly resistive phase. This behavior is in agreement with prior studies in which cooling below ⁓180 K drives the transition from the NCCDW to the insulating CCDW phase^39–41,44^. The inset in Fig. 2b highlights the low-current regime at low temperatures, where a switching-like behavior appears, with current increasing significantly at higher voltages. This non-linear response indicates a barrier-dominated transport mechanism, consistent with the formation of an insulating CCDW channel with very low carrier density, together with non-ideal Au/1T-TaS₂ contacts that become increasingly resistive at low temperature.

The sharp increase in resistance below 180 K is more clearly seen in Fig. 2c which presents the resistance-voltage (*R-V*) plot extracted from the *I-V* data. Two distinct operation regimes spanning several orders of magnitude in resistance are evident : a low-resistance regime (180 K ≤ *T* ≤ 298 K) corresponding to the NCCDW phase and an ultra-high resistance regime (77 K ≤ *T* ≤ 180 K) associated with the CCDW phase, where the superlattice is fully developed in the David-star configuration, resulting in insulating behavior. In the CCDW regime, the resistance gradually decreases by 2–3 orders of magnitude at higher voltages but remains much larger than in the NCCDW phase, indicating a field-assisted enhancement of conduction within a still highly resistive, barrier-dominated state rather than a complete transition back to a metallic phase. This behavior is qualitatively different from the well reported switching-like operation observed in 1T-TaS_2_ crystals, where the application of a threshold electric field induces a volatile phase transition either from the CCDW phase to the NCCDW phase at low temperatures or from the NCCDW phase to the metallic ICCDW phase at room temperature, both accompanied by a sharp increase in current values^39^. In our case, the voltage-induced transition to a more conductive state within the ultra-high resistance CCDW phase takes place in a less abrupt way (observed also in the *I-V* curves, Fig. 2b) and the final conductive state remains significantly more resistive than the NCCDW phase (Fig. 2c). This weaker effect may originate from local Joule heating due to the current and/or from contact-related phenomena^52^, consistent with reports showing that thermally driven, bias-induced changes in CDW order can occur in thin-film 1T-TaS₂ devices under electrical excitation^47^.

After completing the cooling-cycle measurements, the devices were left to return naturally to room temperature (without external heating). The next day, they were vigorously cooled (4–5 K min^− 1^) to 77 K and *I-V* measurements were repeated upon gradual heating. Remarkably, Fig. 2d shows that in this heating cycle the device retains metal-like behavior even in the temperature range normally associated with insulating CCDW phase (77–180 K), with current values comparable to those observed in the NCCDW phase for above 180 K during the cooling cycle (Fig. 2b). In this fast-cooled state, the low-bias *I–V* curves remain nearly linear down to 77 K, despite using the same Au contact geometry as in the gradual-cooling cycle, indicating that the pronounced non-linearity observed after gradual cooling originates primarily from the formation of the insulating CCDW state with strongly reduced carrier density, rather than from contact effects alone.

**Fig. 3:**
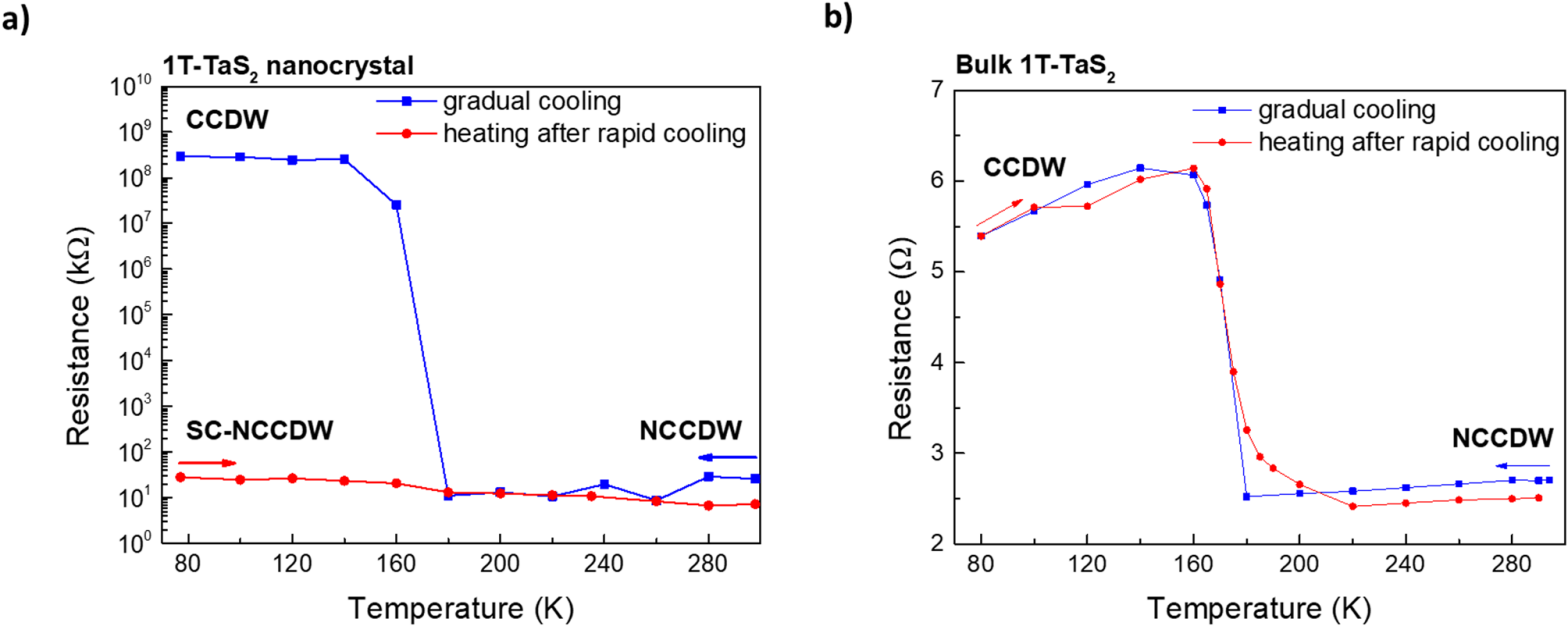
Temperature-dependent resistance of 1T-TaS₂ nanocrystals and bulk crystals. **a** Resistance–temperature (*R–T*) curves of 1T-TaS₂ nanocrystals recorded during gradual cooling (blue square and line) and warming cycle (red circles and line) after rapid cooling. After rapid cooling, the resistance is dramatically decreased, indicating the emergence of super-cooled NCCDW phase. **b** *R–T* characteristics of bulk 1T-TaS₂ crystals recorded during gradual cooling (blue square and line) and warming cycle (red circles and line) after abrupt heating showing the characteristic and reversible NCCDW–CCDW transition near 180 K.

To highlight the emergence of this low-temperature metallic state after rapid cooling, Fig. 3a compares the resistance vs. temperature during gradual cooling and during heating after the vigorous cooling, using a relatively low applied voltage of 0.4 V, at which the *I–V* response is nearly linear in the NCCDW and SC-NCCDW regimes and, in the CCDW phase, still large enough to provide measurable current for a consistent comparison between cooling protocols. During gradual cooling, the resistance exhibits the expected abrupt increase at 180 K, indicating the NCCDW to-CCDW phase transition. In stark contrast, at the beginning of the heating cycle immediately after rapid cooling, the resistance is impressively low at 77 K and remains nearly constant up to ~ 300 K, on the order of the NCCDW resistance. These results indicate that vigorous cooling alters the phase transition pathway by suppressing the formation of the insulating CCDW domains, giving rise to a metastable super-cooled (SC) NCCDW phase, as previously reported by Yoshida et al.^44^.

The influence of crystal thickness on temperature-induced phase transitions was explored by investigating the transport properties of a thick flake (bulk-like 1T-TaS_2_) under the same cooling and heating protocols. The *I-V* curves provided in Supplementary Information, Figure S3 in Supplementary Information exhibits higher linearity (in the near-zero voltage region) compared to nanocrystals consistent with reduced influence of contact effects in larger samples. Figure 3b demonstrates the resistance of bulk 1T-TaS_2_ extracted from the linear part of *I-V* curves as a function of temperature. Here, the typical NCCDW-to-CCDW transition at 180 K is observed upon gradual cooling, and the reverse CCDW-to-NCCDW transition occurs during heating, even after rapid cooling. Thus, in bulk 1T-TaS₂, the phase transition pathway is independent of cooling rate, in agreement with previous reports that the metastable SC-NCCDW phase arises only in thin crystals subjected to rapid cooling^44^. Such thickness-dependent sensitivity is consistent with reports that CDW depinning and switching behaviour become increasingly kinetic in few-layer and nanocrystal regimes^49^.

Our results thus provide further evidence supporting the generation of metastable states at low temperatures with decreased resistance induced by rapid cooling, consistent with the scenario proposed by Yoshida et al.^44^. To elucidate the microscopic origin of the SC-NCCDW phase, we performed temperature-resolved single-crystal XRD and HR-TEM measurements on exfoliated nanocrystals. These structural analyses, presented in the following sections, reveal characteristic modifications of the lattice and domain structure that accompany the stabilization of the super-cooled phase, providing new insights into the interplay between cooling dynamics and CDW ordering in 1T-TaS₂.

### Single-crystal XRD: structural investigation of phase transitions upon cooling

**Fig. 4:**
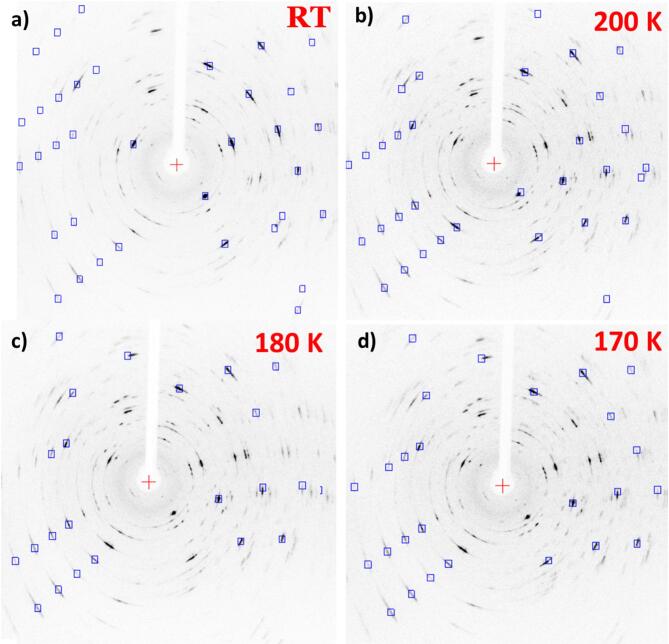
SC-XRD patterns of a thin 1T-TaS₂ crystal upon gradual cooling: **a** RT, **b** 200 K, **c** 180 K, and **d** 170 K. Sharp Bragg spots aligned with the predicted reflections (blue squares) demonstrate high crystallinity. Upon cooling, the reflections become slightly sharper and more intense, consistent with enhanced lattice ordering as the system approaches the low-temperature commensurate regime.

The structural evolution of exfoliated 1T-TaS₂ nanocrystals was examined by temperature-dependent single-crystal XRD (SC-XRD) measurements using two distinct cooling protocols: a gradual, stepwise cooling from room temperature to 170 K and a rapid single-step cooling (RT → 170 K) comparable to the fast-cooling conditions used in the electrical transport measurements (see Methods for details). Figures 4(a–d) display diffraction patterns of a thin 1T-TaS₂ crystal recorded upon gradual cooling from room temperature (RT) to 170 K. At all temperatures, the diffraction patterns exhibit sharp, well-defined Bragg spots that match the calculated reflections (blue squares, Fig. 4), confirming the preservation of single crystallinity and the absence of polycrystalline features, as indicated by the lack of Debye–Scherrer rings. Upon cooling, the reflections become slightly sharper and more intense, consistent with lattice relaxation and ordering as the system approaches the low-temperature commensurate regime.

**Fig. 5:**
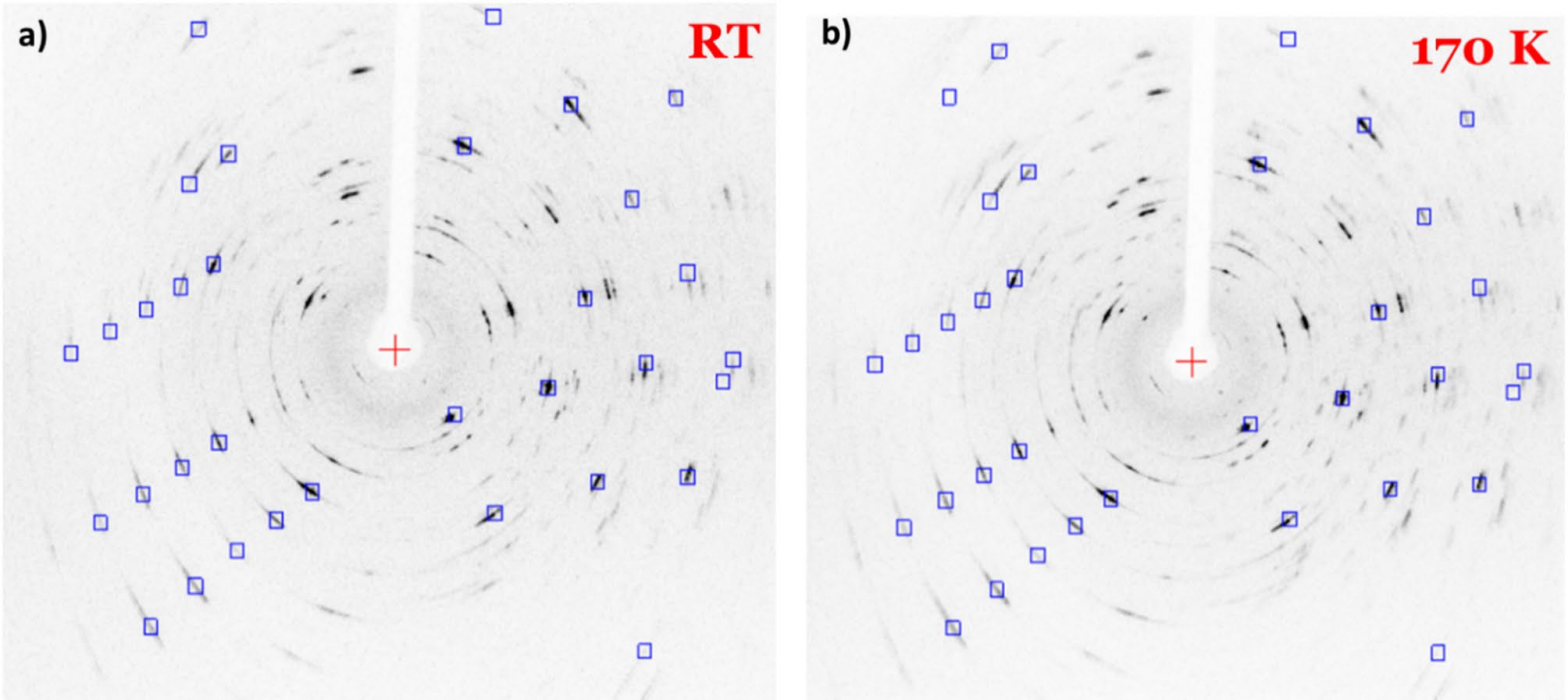
SC-XRD patterns of a thin 1T-TaS₂ crystal after rapid cooling: **a** room temperature (RT) and **b** 170 K. The diffraction patterns remain nearly identical upon cooling, with Bragg reflections matching the predicted positions (blue squares) and no discernible structural modification, indicating that rapid quenching suppresses the lattice reconstruction typically associated with the NCCDW → CCDW transition.

In contrast, single-crystal XRD patterns collected at RT after natural heating and at 170 K following rapid cooling appear nearly identical, indicating that the crystal structure remains essentially unchanged upon cooling under non-equilibrium conditions (Fig. 5a and b). The absence of any discernible structural modification suggests that rapid quenching kinetically inhibits the lattice reconstruction that normally accompanies the NCCDW→CCDW transition. As a result, the system retains a configuration closely resembling the high-temperature NCCDW phase even at low temperature. This behavior correlates with electrical transport measurements, where the sharp resistance increase typically observed during the NCCDW→CCDW transition is suppressed. Instead, the resistance remains low and nearly temperature-independent, indicative of a metallic-like state stabilized by rapid cooling—namely, the super-cooled NCCDW phase.

**Fig. 6:**
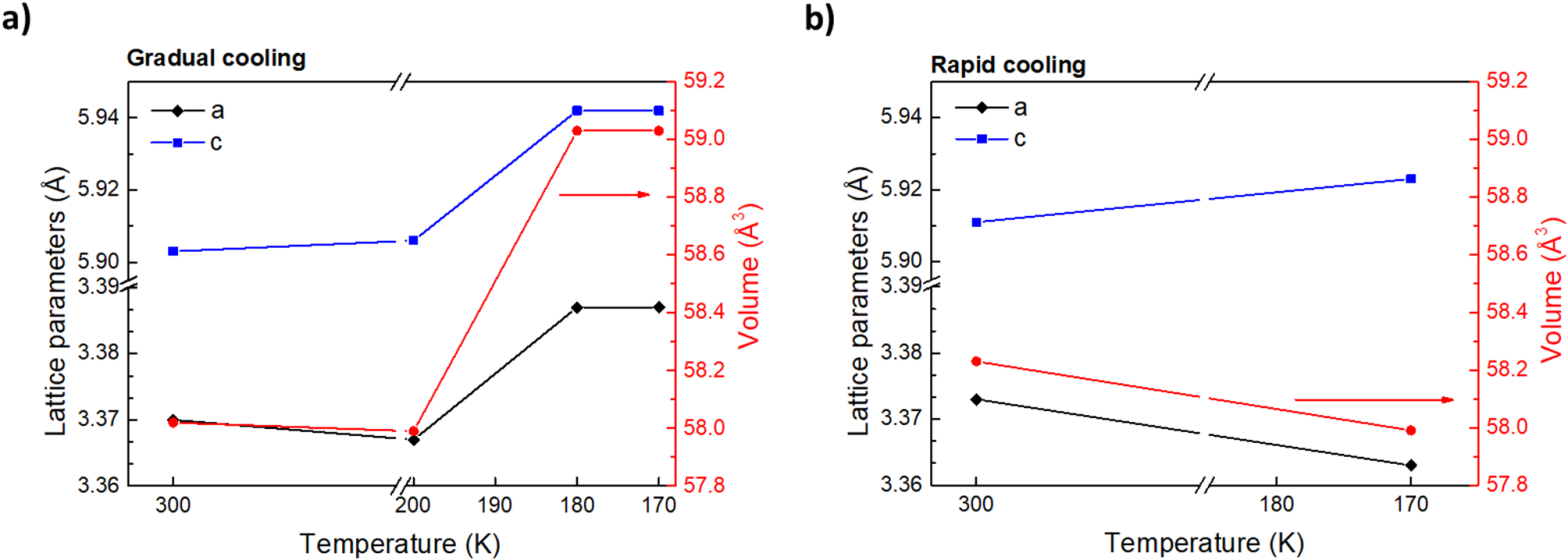
Temperature-dependent lattice parameters and unit-cell volume of thin 1T-TaS₂ crystals. Lattice parameters a and c (left y-axis) and the corresponding unit-cell volume (right y-axis) were extracted from single-crystal XRD measurements as a function of temperature. **a** Under gradual cooling, both a and c exhibit an abrupt expansion below ~ 200 K, leading to a noticeable increase in unit-cell volume, indicative of the transition from NCCDW to the CCDW phase. **b** In contrast, under rapid cooling, this expansion is strongly suppressed, and the overall volume remains almost unchanged, consistent with kinetic stabilization of a super-cooled NCCDW state.

The determination of lattice parameters provides crucial insight into the crystal’s structural response under different cooling regimes. Figure 6 presents the temperature-dependent evolution of the in-plane (a) and out-of-plane (c) lattice constants, as well as the unit-cell volume, as extracted from SC-XRD. In the case of gradual cooling (Fig. 6a), the lattice constants exhibit anisotropic thermal behavior between RT and 200 K, with a slight contraction of the in-plane parameter (a = 3.370 → 3.367 Å) and a minimal expansion of the out-of-plane parameter (c = 5.903 → 5.906 Å), leaving the unit-cell volume nearly unchanged (≈ 58.0 Å³). Upon further cooling to 180 K, however, both a and c increase abruptly (a = 3.387 Å, c = 5.942 Å), leading to a large expansion of the unit cell (ΔV ≈ + 1.0 Å³). This sudden increase could be linked to the NCCDW→CCDW transition, where the formation of “David-star” clusters introduces periodic lattice distortions that expand the crystal framework despite decreasing temperature.

In contrast, samples subjected to rapid cooling after natural heating to RT exhibit a markedly different response (Fig. 6b). At RT, the unit cell is slightly expanded compared to the initial RT state (a = 3.373 Å, c = 5.911 Å), reflecting residual strain from prior gradual cooling. Upon further cooling to 170 K, however, the lattice does not undergo the expected expansion but instead contracts slightly (a = 3.363 Å, c = 5.923 Å, V = 57.99 Å³). This suppression of lattice expansion implies that the cooperative distortions required for CCDW formation are kinetically hindered and instead the system is stabilized in a metastable NCCDW-like configuration.

These structural observations correlate directly with the electronic transport results. During gradual cooling, the pronounced lattice expansion near 180 K coincides with the sharp increase in resistance, reflecting the transition to the insulating CCDW state. In contrast, rapidly cooled samples, which avoid this lattice expansion, maintain metallic-like conductivity down to low temperatures, consistent with the stabilization of a metastable, super-cooled NCCDW-like phase. This clear correspondence between lattice behavior and transport measurements underscores how rapid cooling kinetically hinders the CCDW transition, effectively freezing the system in a metallic state.

To further highlight the impact of reduced dimensionality on the structural and electronic properties of 1T-TaS₂, we performed SC-XRD measurements on thick crystals following the same cooling protocols applied to thin flakes. Unlike the thin crystals, which exhibit sharp diffraction spots indicative of high crystallinity, the thicker samples display pronounced Debye–Scherrer–type rings under both gradual and rapid cooling, reflecting increased mosaicity and structural disorder (Figs. S4 and S5, Supplementary Information). This structural difference has direct consequences on the electronic behavior of 1T-TaS_2_ crystals: rapid cooling of thin flakes results in metal-like conductivity down to low temperatures while bulk crystals undergo the conventional NCCDW → CCDW transition and become insulating even after rapid quenching. Furthermore, analysis of the lattice parameters in thick crystals shows a noticeable unit-cell expansion at low temperatures (Fig. S6, Supplementary Information), consistent with the formation of “David-star” clusters, similar to the behavior observed in nanocrystals. Overall, these results demonstrate that reduced dimensionality facilitates the kinetic stabilization of non-equilibrium phases in 1T-TaS₂, linking structural confinement to the emergence of metallic transport in thin flakes. This dimensionality dependence is consistent with bulk STM/XRD studies by Riffle et al., who reported that varying the cooling rate from slow to ultrafast in macroscopic 1T-TaS₂ crystals generally preserves the CCDW superlattice at low temperature^50^. Together with our observation that bulk-like flakes also retain the conventional NCCDW→CCDW transition under rapid cooling, this highlights reduced dimensionality as the key factor enabling kinetic suppression of CCDW formation and stabilization of the SC-NCCDW state.

### HR-TEM investigation: insights into the microscopic nature of SC-NCCDW phase

Even though single-crystal XRD provides valuable insight into the lattice evolution and phase stabilization in both thin and bulk 1T-TaS₂ crystals, it offers limited spatial resolution for directly visualizing the local atomic arrangements associated with the SC-NCCDW phase. To explore the atomic-scale configuration and better understand how rapid cooling kinetically stabilizes this metastable state we carried out high-resolution transmission electron microscopy (HR-TEM) measurements on thin nanocrystals. More specifically, two distinct states were examined: (i) the room-temperature (RT) state, corresponding to the nearly commensurate (NC) CDW phase, and (ii) the fast-cooled (FC) state, obtained by quenching the sample to 77 K followed by rapid heating back to RT (details in Methods). Therefore, the micrographs corresponding to the FC state probe the configuration that is retained after the fast quench to 77 K and subsequent reheating to RT, rather than an in situ low-temperature state.

**Fig. 7:**
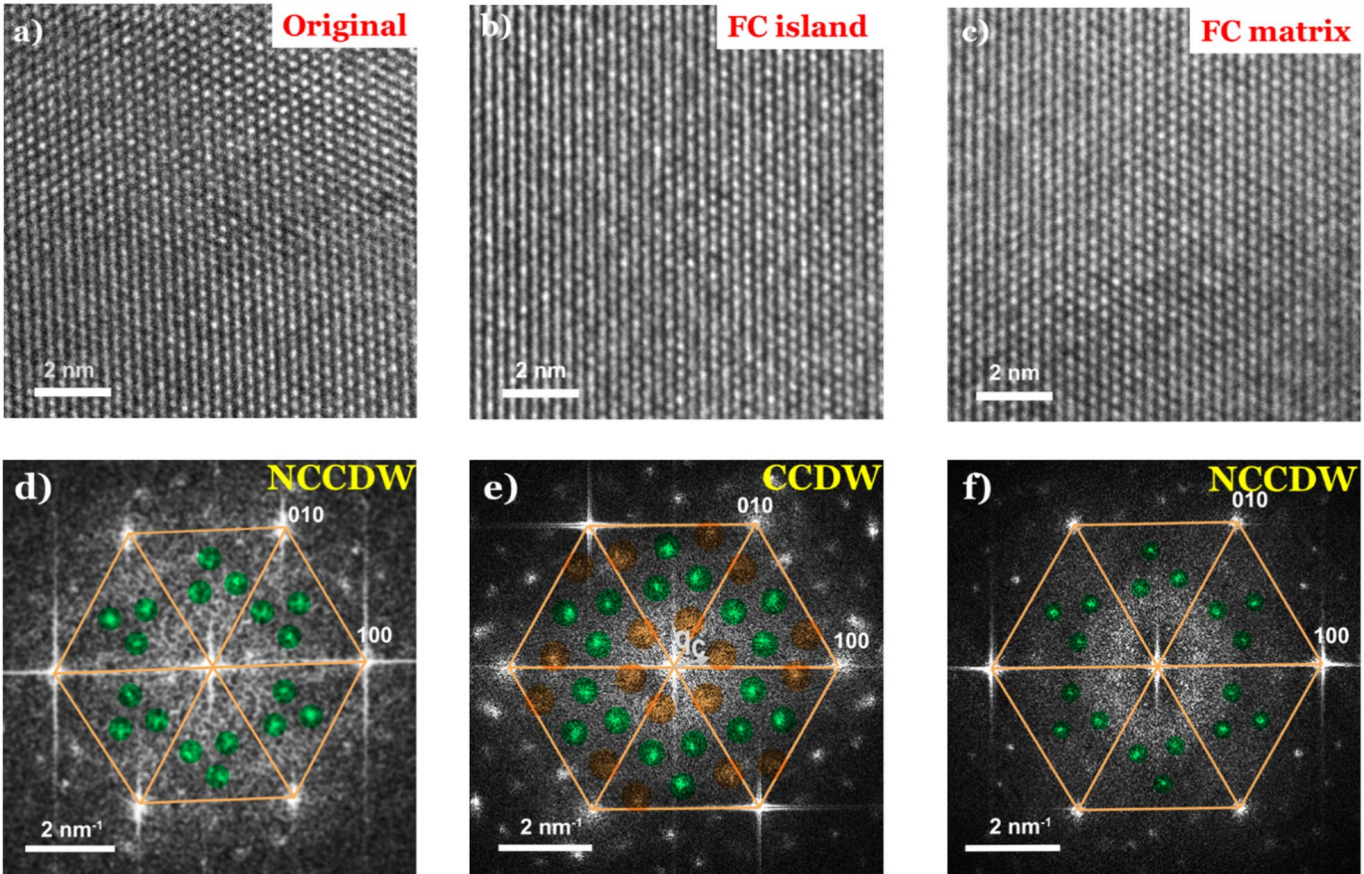
High-resolution transmission electron microscopy (HR-TEM) investigation of 1T-TaS₂ and CDW phases. **a** HRTEM image of original 1T-TaS₂ at room temperature (RT), **b** the fast-cooled (FC) island region, and **c** the FC matrix region. **d**–**f** Corresponding fast Fourier transform (FFT) patterns of panels (a–c), respectively. The weaker diffraction spots marked by green and orange circles indicate the nearly commensurate charge-density-wave (NC-CDW) and commensurate charge-density-wave (C-CDW) states, respectively. The angle between the superlattice vector **q**_c_ and the primary lattice vector **g**₁₀₀ is 13.9°.

Extensive examination of the FC sample revealed the coexistence of nanoscale (10–30 nm) islands (domains) with atomic arrangements distinct from the surrounding the 1T-TaS₂ matrix. Representative HR-TEM images of the RT sample, an FC island, and the FC matrix are shown in Fig. 7(a–c), with their corresponding Fast Fourier Transforms (FFTs) in Fig. 7(d–f) using colored overlays to mark the CDW satellites. For completeness, the original FFT patterns without schematics are provided in Supplementary Information, Fig. S7. Indexing of the FFTs confirms that all regions are oriented along the [001] crystallographic axis. The {100} reflections correspond to the basic lattice constant of a ≈ 0.336 nm.

**Fig. 8:**
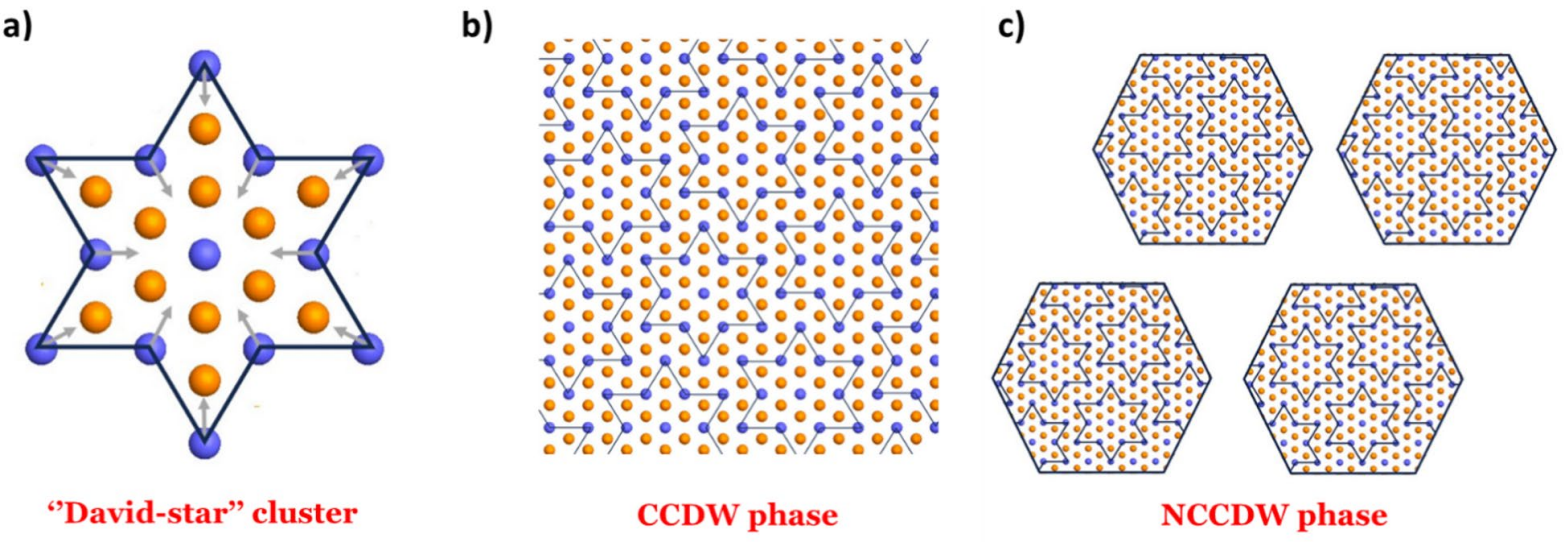
Schematic representations of charge-density-wave (CDW) structures in 1T-TaS₂. **a** Illustration of the twelve Ta atoms displaced toward a central thirteenth Ta atom, forming the characteristic “David-star” cluster. **b** Atomic arrangement of Ta atoms in the commensurate CDW (CCDW) phase. **c** Atomic arrangement of Ta atoms in the nearly commensurate CDW (NCCDW) phase.

It is well established^3,5,31,53–55^ that strong electron–phonon coupling in 1T-TaS₂ leads to periodic lattice distortions associated with CDWs. Below ~ 180 K, the ground state is the CCDW phase, in which twelve Ta atoms contract toward a central thirteenth Ta to form the characteristic “David-star” clusters (schematically shown in Fig. 8a). These clusters produce the CCDW superlattice with periodicity *λ*_*c*_ *= √13a*, rotated by *φ*_*c*_ *≈ 13.9°* relative to the underlying atomic lattice as shown in Fig. 8b. At room temperature, however, 1T-TaS₂ adopts the NCCDW phase, where the above ordering is preserved in comprising tens of stars, as shown in Fig. 8c.

The FFT of the original (RT) sample (Fig. 7d) displays the expected satellite reflections, marked by green circles, appearing as three additional diffraction spots within each triangle formed by adjacent {100} reflections and the central beam. These features are consistent with the NCCDW phase^16^. In contrast, the FC sample exhibits nanoscale regions that locally adopt a fully commensurate CDW structure. This is evident from the six weak spots observed in each triangle of the FFT shown in Fig. 7e, marked by orange and green circles, characteristic of the CCDW superlattice^16^. These islands reside inside the 1T-TaS_2_ matrix that retains the NCCDW state as evidenced as confirmed by the FFT shown in Fig. 7f where three diffraction spots, marked by green circles, are observed as in original (RT) state^16^. We note that this mixed CCDW/NCCDW diffraction pattern was reproducibly observed across several independently prepared fast-cooled specimens, confirming that it is an intrinsic quenched configuration rather than an artifact of a single cooling–reheating cycle.

Taken together, these results demonstrate that rapid cooling drives 1T-TaS₂ into a metastable mixed state, where nanoscale CCDW islands are embedded within an NCCDW background. This heterogeneous super-cooled configuration provides direct structural evidence for the coexistence of phases that underpin the anomalous metallic transport observed in electrical measurements.

## Discussion

In this work, we systematically investigated the electrical and structural properties of the low-temperature super-cooled nearly commensurate charge-density-wave (SC-NCCDW) phase in 1T-TaS₂ nanocrystals induced by rapid cooling. Temperature-dependent current–voltage (*I–V*) measurements of nanocrystal devices, fabricated via electron-beam lithography, reveal the characteristic transition from the low-resistance nearly commensurate CDW (NCCDW) phase to the high-resistance commensurate CDW (CCDW) phase upon gradual cooling, manifested by a sharp resistance increase around 180 K. In contrast, after vigorous cooling to 77 K, the *I–V* characteristics remain metallic-like over the entire temperature range, with resistance values comparable to those of the room-temperature NCCDW phase. This behavior evidences the formation of a super-cooled NCCDW phase, a metastable metallic state stabilized by rapid cooling. Electrical characterization of bulk crystals, on the other hand, shows that the CDW transition sequence is independent of cooling rate, underscoring the crucial role of reduced dimensionality in governing non-equilibrium behavior.

Motivated by these results, we conducted temperature-dependent single-crystal X-ray diffraction (SC-XRD) and high-resolution transmission electron microscopy (HR-TEM) to uncover the microscopic origin of the super-cooled state. XRD measurements revealed that 1T-TaS₂ nanocrystals maintain high crystallinity under both gradual and rapid cooling, as evidenced by sharp Bragg reflections without polycrystalline features. The extraction of lattice parameters *a* and *c* reveal that both in-plane and out-of-plane distances abruptly increase at 180 K upon gradual cooling, leading to a clear unit-cell expansion. This expansion is attributed to the lattice distortion taking place during the NCCDW to CCDW phase transition. On the contrary, upon rapid cooling rapid parameter *a* decreases slightly while parameter *c* increases slightly leaving the unit-cell volume almost unchanged, indicating the suppression of lattice distortion which normally leads to CCDW phase. These structural observations correlate directly with the electronic transport results and the emergence of the SC-NCCDW phase after rapid cooling.

HR-TEM investigation provided complementary atomic-scale insight into this metastable configuration. The HR-TEM and corresponding FFT analyses reveal that the fast-cooled samples contain nanoscale (10–30 nm) islands exhibiting CCDW structure embedded within a matrix that retains the nearly NCCDW ordering. The coexistence of these nanoscale CCDW domains and the surrounding NCCDW regions demonstrates that rapid cooling drives 1T-TaS₂ into an intermixed SC-NCCDW state rather than into a uniform phase.

Taken together, the combined electrical, XRD, and HR-TEM results establish that rapid cooling kinetically suppresses the CCDW transition and stabilizes a metastable, super-cooled NCCDW phase with metallic transport properties in 1T-TaS₂ nanocrystals. Our findings provide the first direct structural–electronic correlation linking the suppression lattice distortions with the emergence of metallic behavior in the SC-NCCDW phase. By uncovering the microscopic origin of this metastable phase—arising from the kinetic freezing of CDW domain evolution and the coexistence of nanoscale commensurate islands within a nearly commensurate matrix—this work provides new insight into the SC-NCCDW state in 1T-TaS₂ and offers a basis for controlling non-equilibrium electronic phases in low-dimensional materials.

## Methods

*Samples preparation and device fabrication.* 1T-TaS_2_ nanocrystals were mechanically exfoliated from a bulk commercial crystal (Hq graphene) and transferred on SiO_2_/Si substrates prepatterned with square (150 μm x150µm) gold (Au) pads and alignment markers. The 1T-TaS_2_ nanocrystals were connected to the Au pads via 500 nm-wide Au wires aligned and patterned using electron beam lithography (RAITH EBPG 5000plus) at 100 kV, followed by sequential thermal deposition of titanium (Ti) and gold (Au) (5 nm/85nm).

The exposed patterns were designed on Klayout following sample inspection and careful coordinate transformation of topographical data. Proximity effect correction and lithographic data preparation (Beamer, Genisys) were applied. The 340nm thick bi-layer formed by MMA (AR-P 617.08) as bottom layer and 70nm-thick PMMA (996k) as top layer, was developed in a MIBK: IPA (1:3) solution for 60”, with a typical dose to clear equal to 370±10µC/cm^2^.

*Characterization techniques*. 5 × 5 µm^2^ AFM images of exfoliated crystals were recorded using an NT-MDT AFM system in tapping operation mode. SEM images of the exfoliated nanocrystals were recorded with a JEOL 7401f FESEM. Raman spectra were acquired using a LabRAM Soleil Horiba Raman microscope with 532 nm laser excitation, focused by a100 (NA = 0.9) objective, while a laser diode emitting at 532 nm.

*Powder X-ray diffraction (XRD)* measurements were performed using a Rigaku SmartLab diffractometer in θ/θ geometry with Bragg–Brentano optics. The instrument was equipped with a secondary beam pyrolytic graphite monochromator and utilized Cu Kα radiation (Cu Kα₁ = 1.54060 Å, Cu Kα₂ = 1.54439 Å). The thick exfoliated sample was analyzed under generator settings of 40 kV and 35 mA, using slits of 2/3°, 2/3°, and 0.6°. Data were collected over the 2θ range 2.0–110.0.0.0^o^ with a step size of 0.03° and a counting time of 5 s per step.

*Single-crystal X-ray diffraction (SC-XRD)* measurements were carried out on a Rigaku R-AXIS SPIDER image plate diffractometer using graphite-monochromated Mo Kα radiation. The system was equipped with an X-Stream 2000 low-temperature unit (nitrogen generator with He compressor**).** Data collection (ω-scans) was conducted using the CrystalClear-SM software package [Version 1.40 rc7, Rigaku/MSC Inc., The Woodlands, Texas, USA; https://rigaku.com/products/crystallography]. Measurements were performed at selected temperatures between 170 K and room temperature (RT). In the gradual-cooling protocol, the crystal was cooled in small temperature steps (20 K per step), with each step completed within approximately 10 min and followed by thermal stabilization keeping an average cooling rate of approximately 2 K min⁻^1^. In the rapid-cooling cycle, the crystal was first returned to RT and then cooled directly to 170 K in a single step at the maximum rate of the cryosystem, giving an effective rate on the order of 4–5 K min⁻¹. Plots of the structure were drawn using the Diamond 3 program package [DIAMOND – Crystal and Molecular Structure Visualization, Version 3.1, Crystal Impact, Rathausgasse 30, 53111, Bonn, Germany; https://www.crystalimpact.com/diamond].

*HR-TEM* study was undertaken using a Thermo Fisher Talos F200i TEM operating at 200 kV. The TEM specimens were prepared by drop casting 10µL of 1T-TaS_2_ isopropanol suspension onto a holey carbon support TEM grid inside a glove box under an Ar inert atmosphere. Following evaporation of the suspension the TEM specimen was fast-cooled to 77 K within less than 60 s and then rapidly re-heated to room temperature within less than 60 s (FC) while another specimen was retained at room temperature denoted as original sample. Specimen FC was inserted in the TEM within a five-minutes time interval and was examined within the same day (within a few hours). The mixed CCDW/NCCDW contrast described in the Results was observed reproducibly on multiple FC grids prepared in this manner. Fast Fourier Transformations (FFTs) were calculated from HR- TEM images utilizing DigitalMicrograph software [Version 1.71.38, Gatan Inc., Pleasanton, CA, USA; https://www.gatan.com/products/tem-analysis/gatan-microscopy-suite-software], while gamma correction was applied to the FFTs in order to improve visibility of the weaker diffraction spots using the same software.

*Two-probe current–voltage electrical measurements* of exfoliated 1T-TaS_2_ nanocrystals were carried out using a Janis ST-500–2 cryogenic probe station within the temperature range of 77 K-RT.

## Supplementary Information

Below is the link to the electronic supplementary material.

Supplementary Material 1

## Data Availability

Data is provided within the manuscript or supplementary information files. The datasets used during the current study are also available from the corresponding author on reasonable request.

## References

[1] Chen, H. et al. The recent progress of two-dimensional transition metal dichalcogenides and their phase transition. *Crystals* **12**, 1381 (2022).

[2] Fei, Y., Wu, Z., Zhang, W. & Yin, Y. Understanding the Mott insulating state in 1T-TaS_2_ and 1T-TaSe_2_. *AAPPS Bull.* **32**, 20 (2022).

[3] Sipos, B. et al. From Mott state to superconductivity in 1T-TaS_2_. *Nat. Mater.* **7**, 960–965 (2008). 18997775 10.1038/nmat2318

[4] Pan, X. et al. Controllable synthesis and charge density wave phase transitions of two-dimensional 1T-TaS_2_ crystals. *Nanomaterials* **13**, 1806 (2023). 37299709 10.3390/nano13111806 PMC10254911

[5] Hovden, R. et al. Atomic lattice disorder in charge-density-wave phases of exfoliated dichalcogenides (1T-TaS_2_). *Proc. Natl. Acad. Sci. U.S.A.* **113**, 11420–11424 (2016). 27681627 10.1073/pnas.1606044113 PMC5068312

[6] Wilson, J. A., Di Salvo, F. J. & Mahajan, S. Charge-density waves and superlattices in the metallic layered transition metal dichalcogenides. *Adv. Phys.* **24**, 117–201 (1975).

[7] Joshi, J. et al. Short-range charge density wave order in 2 H – T a S 2. *Phys. Rev. B*. **99**, 245144 (2019).

[8] Wayman, C. M., Bhadeshia, H. K. & D, H. Phase transformations, nondiffusive. In *Physical Metallurgy* Vol. 2 (eds Cahn, R. W. & Haasen, P.) 1507–1554 (North-Holland, 1996).

[9] Hwang, J. et al. Charge density waves in two-dimensional transition metal dichalcogenides. *Rep. Prog Phys.* **87**, 044502 (2024). 10.1088/1361-6633/ad36d3 38518359

[10] Castro Neto, A. H. Charge density wave, superconductivity, and anomalous metallic behavior in 2D transition metal dichalcogenides. *Phys. Rev. Lett.* **86**, 4382–4385 (2001). 11328180 10.1103/PhysRevLett.86.4382

[11] Leroux, M., Cario, L., Bosak, A. & Rodière, P. Traces of charge density waves in NbS_2_. *Phys. Rev. B* **97**, 195140 (2018).

[12] Sugawara, K. et al. Unconventional charge-density-wave transition in monolayer 1T -TiSe_2_. *ACS Nano* **10**(1), 1341–1345 (2016). 26624791 10.1021/acsnano.5b06727

[13] Dai, J. et al. Microscopic evidence for strong periodic lattice distortion in two-dimensional charge-density wave systems. *Phys. Rev. B* **89**, 165140 (2014).

[14] McMillan, W. L. Landau theory of charge-density waves in transition-metal dichalcogenides. *Phys. Rev. B* **12**, 1187–1196 (1975).

[15] Lu, H. & Guo, W. Tunable charge density wave phases in transition metal dichalcogenides heterostructures. *Phys. Rev. B* **108**, 224103 (2023).

[16] Tsen, A. W. et al. Structure and control of charge density waves in two-dimensional 1T-TaS_2_. *Proc. Natl. Acad. Sci. U.S.A.* **112**, 15054–15059 (2015). 26598707 10.1073/pnas.1512092112 PMC4679066

[17] Wang, G. et al. Atomic visualization of the 3D charge density wave stacking in 1T-TaS_2_ by cryogenic transmission electron microscopy. *Nano Lett.* **23**, 4318–4325 (2023). 37159525 10.1021/acs.nanolett.3c00556

[18] Fazekas, P. & Tosatti, E. Electrical, structural and magnetic properties of pure and doped 1T-TaS_2_. *Philosophical Magazine B* **39**, 229–244 (1979).

[19] Perfetti, L. et al. Time evolution of the electronic structure of 1T -TaS_2_ through the insulator-metal transition. *Phys. Rev. Lett.* **97**, 067402 (2006). 17026203 10.1103/PhysRevLett.97.067402

[20] Petkov, V., Peralta, J. E., Aoun, B. & Ren, Y. Atomic structure and Mott nature of the insulating charge density wave phase of 1T-TaS_2_. *J. Phys. Condens. Matter* **34**, 345401 (2022). 10.1088/1361-648X/ac77cf 35688141

[21] Ma, L. et al. A metallic mosaic phase and the origin of Mott-insulating state in 1T-TaS_2_. *Nat. Commun.* **7**(1), 10956 (2016). 26961788 10.1038/ncomms10956 PMC4792954

[22] Butler, C. J. et al. Mottness versus unit-cell doubling as the driver of the insulating state in 1T-TaS_2_. *Nat. Commun.* **11**(1), 2477 (2020). 32424136 10.1038/s41467-020-16132-9 PMC7235044

[23] Ritschel, T. et al. Orbital textures and charge density waves in transition metal dichalcogenides. *Nat. Phys.* **11**, 328–331 (2015).

[24] Wang, Y. D. et al. Band insulator to Mott insulator transition in 1T-TaS_2_. *Nat. Commun.* **11**(1), 4215 (2020). 32839433 10.1038/s41467-020-18040-4 PMC7445232

[25] Philip, S. S., Neuefeind, J. C., Stone, M. B. & Louca, D. Local structure anomaly with the charge ordering transition of 1 T − TaS 2. *Phys. Rev. B* **107**, 184109 (2023).

[26] Li, L. J. et al. Fe-doping–induced superconductivity in the charge-density-wave system 1T-TaS _2_. *EPL* **97**, 67005 (2012).

[27] Dong, Q. et al. Structural phase transition and superconductivity hierarchy in 1T-TaS2 under pressure up to 100 GPa. *npj Quantum Mater.* **6**, 20 (2021).

[28] Lee, P. A. & Rice, T. M. Electric field depinning of charge density waves. *Phys. Rev. B* **19**, 3970–3980 (1979).

[29] Durham, D. B. et al. Nanosecond structural dynamics during electrical melting of charge density waves in 1 T − TaS 2. *Phys. Rev. Lett.* **132**, 226201 (2024). 38877909 10.1103/PhysRevLett.132.226201

[30] Han, T.-R. et al. Exploration of metastability and hidden phases in correlated electron crystals visualized by femtosecond optical doping and electron crystallography. *Sci. Adv.* **1**, e1400173 (2015). 26601190 10.1126/sciadv.1400173 PMC4640616

[31] Achari, A. et al. Alternating superconducting and charge density wave monolayers within bulk 6R-TaS_2_. *Nano Lett.* **22**, 6268–6275 (2022). 35857927 10.1021/acs.nanolett.2c01851 PMC9373026

[32] Markovic, N., Dohmen, M. A. H. & van der Zant, H. S. J. Tunable charge density wave transport in a current-effect transistor. *Phys. Rev. Lett.* **84**, 534–537 (2000). 11015957 10.1103/PhysRevLett.84.534

[33] de la Torre, A. et al. Dynamic phase transition in 1T-TaS_2_ via a thermal quench. *Nat. Phys.* **21**, 1267–1274 (2025).

[34] Sun, K. et al. Hidden CDW states and insulator-to-metal transition after a pulsed femtosecond laser excitation in layered chalcogenide 1T-TaS_2− x_Se_x_. *Sci. Adv.* **4**, eaas9660 (2018). 30035223 10.1126/sciadv.aas9660 PMC6054513

[35] Laulhé, C. et al. Ultrafast formation of a charge density wave state in 1 T−TaS_2_: Observation at nanometer scales using time-resolved X-ray diffraction. *Phys. Rev. Lett.* **118**, 247401 (2017). 28665649 10.1103/PhysRevLett.118.247401

[36] Shao, D. F. et al. Manipulating charge density waves in 1T−TaS_2_ by charge-carrier doping: A first-principles investigation. *Phys. Rev. B* **94**, 125126 (2016).

[37] Vaskivskyi, I. et al. Controlling the metal-to-insulator relaxation of the metastable hidden quantum state in 1T-TaS_2_. *Sci. Adv.* **1**, e1500168 (2015). 26601218 10.1126/sciadv.1500168 PMC4646782

[38] Stojchevska, L. et al. Ultrafast switching to a stable hidden quantum state in an electronic crystal. *Science* **344**, 177–180 (2014). 24723607 10.1126/science.1241591

[39] Hollander, M. J. et al. Electrically driven reversible insulator–metal phase transition in 1T-TaS_2_. *Nano Lett.* **15**, 1861–1866 (2015). 25626012 10.1021/nl504662b

[40] Zheng, S. et al. Room-temperature electrically driven phase transition of two-dimensional 1T-TaS_2_ layers. *Nanoscale* **9**, 2436–2441 (2017). 28150828 10.1039/c6nr07541j

[41] Yoshida, M. et al. Memristive phase switching in two-dimensional 1T-TaS _2_ crystals. *Sci. Adv.* **1**, e1500606 (2015). 26601295 10.1126/sciadv.1500606 PMC4646809

[42] Geremew, A. K. et al. Bias-voltage driven switching of the charge-density-wave and normal metallic phases in 1T-TaS _2_ thin-film devices. *ACS Nano* **13**, 7231–7240 (2019). 31173685 10.1021/acsnano.9b02870

[43] Yu, Y. et al. Gate-tunable phase transitions in thin flakes of 1T-TaS_2_. *Nat. Nanotechnol.* **10**, 270–276 (2015). 25622230 10.1038/nnano.2014.323

[44] Yoshida, M. et al. Controlling charge-density-wave states in nano-thick crystals of 1T-TaS_2_. *Sci. Rep.* **4**(1), 7302 (2014). 25466764 10.1038/srep07302 PMC4252899

[45] S, S., Kundu, H. K., Vishnubhotla, P. & Bid, A. Hidden electronic phase in strained few-layer 1 T −TaS_2_. *Phys. Rev. Mater.* **5**, 124003 (2021).

[46] Ritschel, T. et al. Pressure dependence of the charge density wave in 1 T -TaS 2 and its relation to superconductivity. *Phys. Rev. B*. **87**, 125135 (2013).

[47] Mohammadzadeh, A. et al. Evidence for a thermally driven charge-density-wave transition in 1T-TaS2 thin-film devices: Prospects for GHz switching speed. *Appl. Phys. Lett.* **118**, 093102 (2021).

[48] Taheri, M. et al. Electrical gating of the charge-density-wave phases in two-dimensional h-BN/1T-TaS2 devices. *ACS Nano* **16**, 18968–18977 (2022). 36315105 10.1021/acsnano.2c07876

[49] Brown, J. O. et al. Current fluctuations and domain depinning in quasi-two-dimensional charge-density-wave 1T-TaS2 thin films. *Appl. Phys. Rev.* **10**, 041401 (2023).

[50] Riffle, J. V. et al. Cooling rate dependence of charge density wave phases in 1T-TaS2 studied by scanning tunneling microscopy and x-ray diffraction. *AIP Adv.* **14**, 105303 (2024).

[51] Zhao, Y. et al. Spectroscopic visualization and phase manipulation of chiral charge density waves in 1T-TaS_2_. *Nat. Commun.* **14**(1), 2223 (2023). 37076513 10.1038/s41467-023-37927-6 PMC10115830

[52] Baraghani, S. et al. Charge-density-wave thin-film devices printed with chemically exfoliated 1T-TaS_2_ ink. *ACS Nano* **16**, 6325–6333 (2022). 35324143 10.1021/acsnano.2c00378

[53] Ishiguro, T. & Sato, H. Electron microscopy of phase transformations in 1T-TaS_2_. *Phys. Rev. B* **44**, 2046–2060 (1991). 10.1103/physrevb.44.2046 9999754

[54] Chang, Y.-Y. et al. Analyzing the microstructure and related properties of 2D materials by transmission electron microscopy. *Appl. Microsc.* **49**, 10 (2019). 33580317 10.1186/s42649-019-0013-5 PMC7809582

[55] Chen, M. et al. Interlayer coupling and the phase transition mechanism of stacked MoS_2_ /TaS_2_ heterostructures discovered using temperature dependent Raman and photoluminescence spectroscopy. *RSC Adv.* **8**, 21968–21974 (2018). 35541734 10.1039/c8ra03436b PMC9081101

