# Supplementary material for: Microscopic insight into the origin of super-cooled NCCDW state in 1T-TaS₂ nanocrystals
Source: Sci Rep. 2026 Mar 25;16:14925. doi: 10.1038/s41598-026-42525-9 (PMC13168586; doi:10.1038/s41598-026-42525-9)
Supplement: Supplementary file 1 — Supplementary Material 1 [file 41598_2026_42525_MOESM1_ESM.docx]

**Supplementary Information**

**Microscopic Insight into the Cooling-Rate–Induced Super-Cooled NCCDW State in 1T-TaS₂ Nanocrystals**

**Georgios Chatzigiannakis^1,2,*^, Anastasia Soultati^1^, Elias Sakellis^1,2^, George Papageorgiou^1^, Nikos Boukos^1^, Vassilis Psycharis^1^, Catherine P. Raptopoulou^1^, Konstantinos Aidinis^3,4^, Spiros Gardelis^2^, Alexander Chroneos^5,6,*^, Maria Vasilopoulou^1,*^**

^1^ Institute of Nanoscience and Nanotechnology, National Center for Scientific Research “Demokritos”, Agia Paraskevi 15341, Athens, Greece.

^2^ Section of Condensed Matter Physics, Department of Physics, National and Kapodistrian University of Athens, Panepistimioupolis, Zografos 15784, Athens, Greece.

^3^ Department of Electrical and Computer Engineering, Ajman University, P.O. Box 346, Ajman, United Arab Emirates.

^4^ Center of Medical and Bio-Allied Health Sciences Research, Ajman, United Arab Emirates.

^5^ Department of Electrical and Computer Engineering, University of Thessaly, 38221, Volos, Greece.

^6^ Department of Materials, Imperial College, London SW7 2AZ, UK.

[^*^g.chatzigiannakis@inn.demokritos.gr](mailto:*g.chatzigiannakis@inn.demokritos.gr)

^*^[m.vasilopoulou@inn.demokritos.gr](mailto:m.vasilopoulou@inn.demokritos.gr)

^*^[alexander.chroneos@imperial.ac.uk](mailto:alexander.chroneos@imperial.ac.uk)

**Figure S1:** Raman spectrum of exfoliated 1T-TaS_2_ nanocrystal.


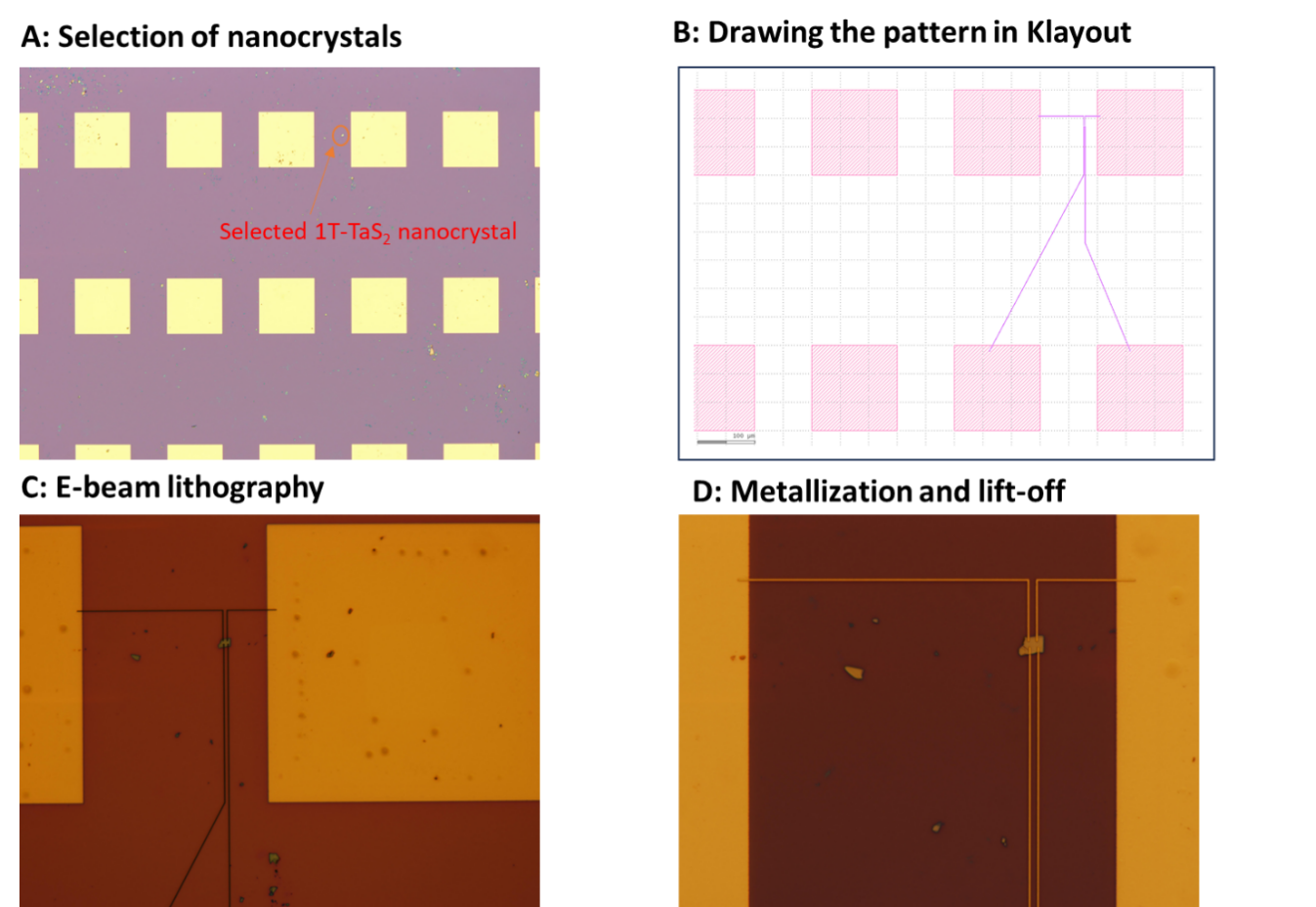


**Figure S2:** Schematic of the process followed for 1T-TaS_2_ crystals selection for e-beam consisted of the following steps: (a) selection of nanocrystals on optical microscope image, (b) patterns drawing using KLayout (c) electron-beam (e-beam) lithography process, (d) thermal deposition of titanium (Ti) and gold (Au) (5nm/85nm).

**
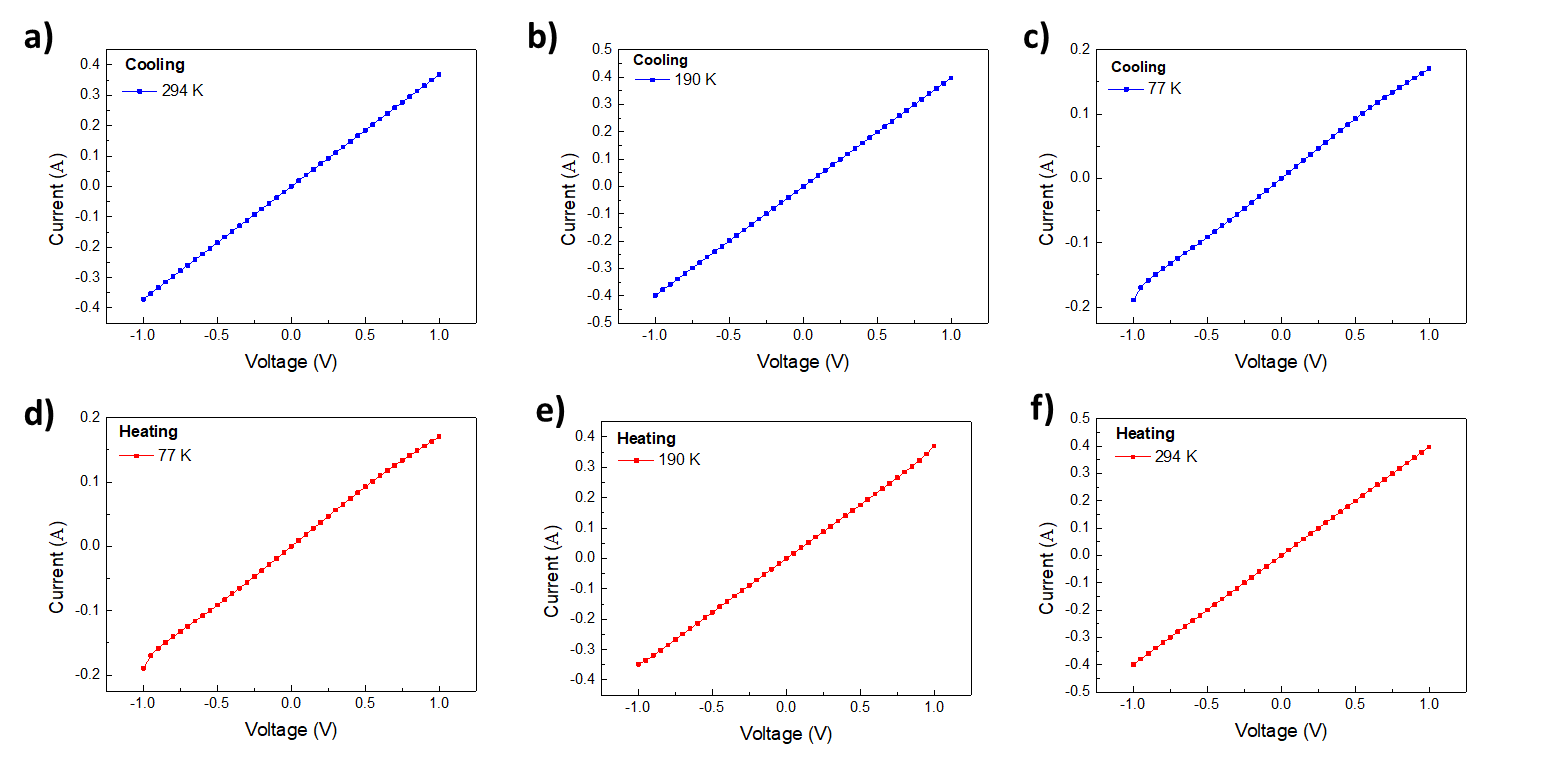
**

**Figure S3:** Current–voltage (*I–V*) curves of thick (bulk) 1T-TaS_2_ crystals measured at selected temperatures during (a–c) cooling and (d–f) subsequent heating. The data show linear and symmetric I–V behavior across all temperatures, indicating ohmic contact and stable electronic transport during both cooling and warming cycles.

**
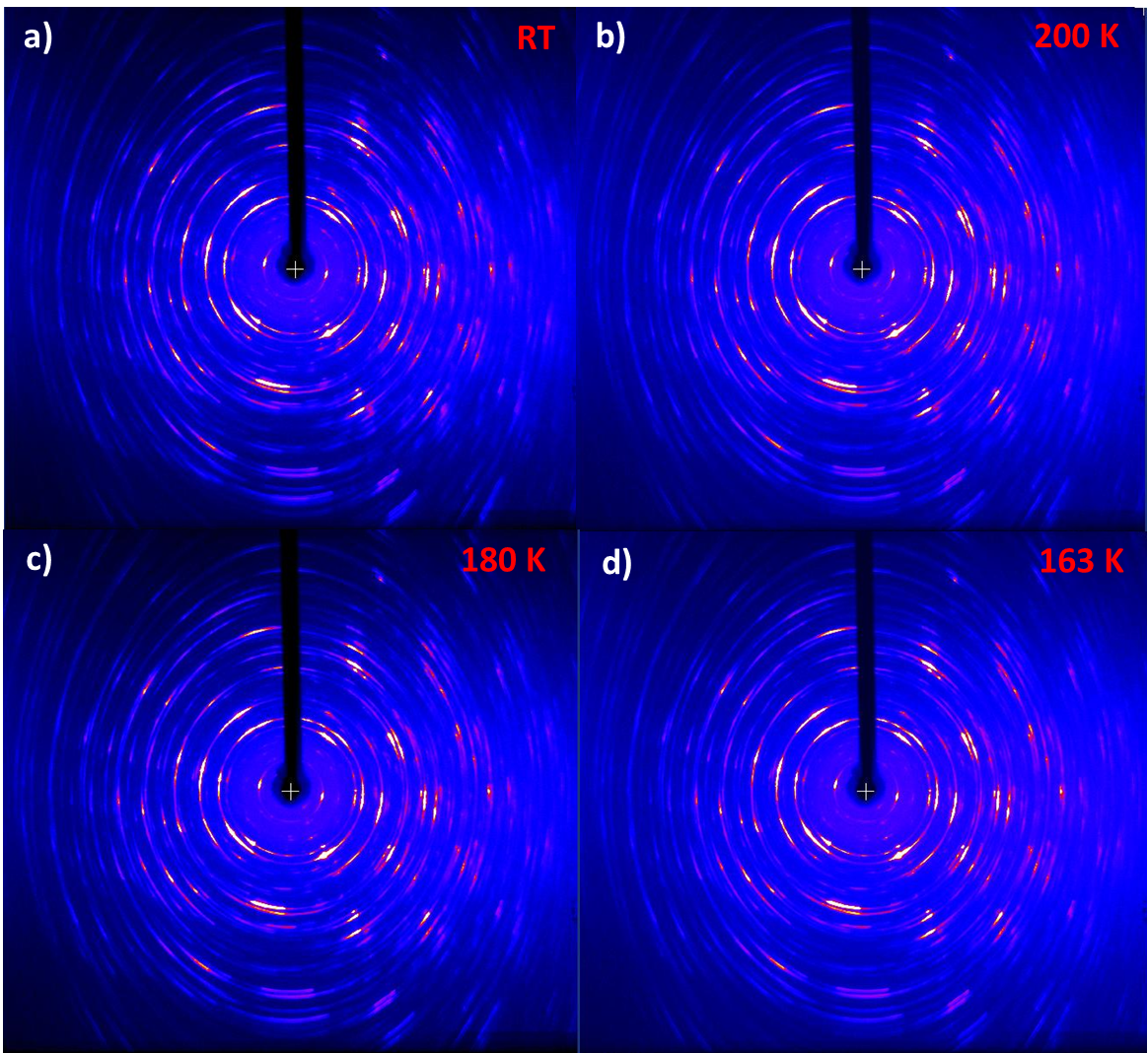
**

**Figure S4:** SC-XRD diffraction patterns collected of thick 1T-TaS_2_ crystals upon gradual cooling at (a) room temperature (RT), (b) 200 K, (c) 180 K, and (d) 163 K. The presence of continuous Scherrer rings indicates that the thick crystals exhibit polycrystalline characteristics.

**
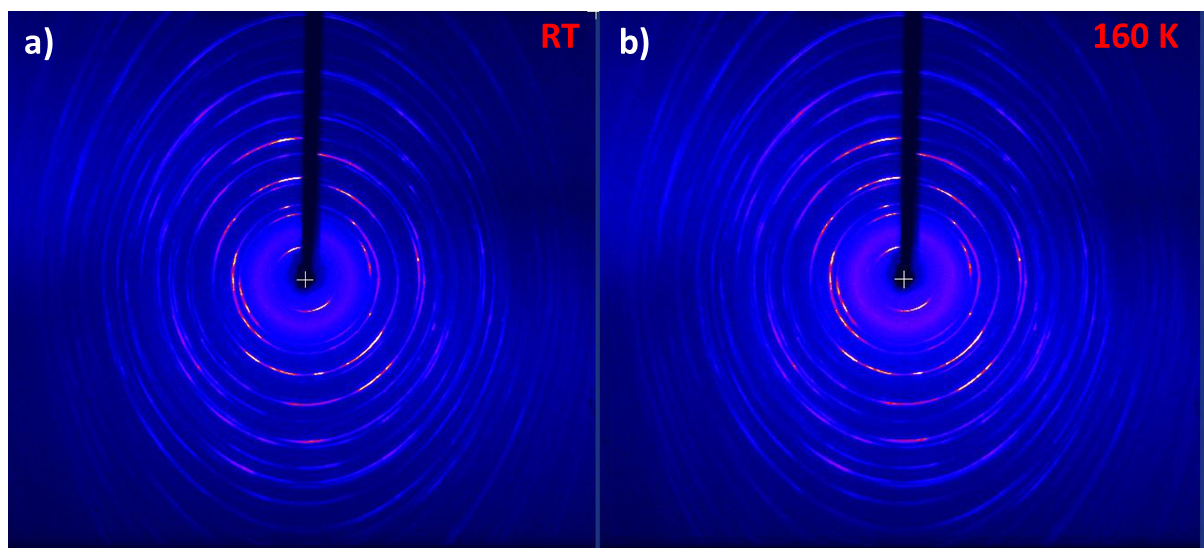
**

**Figure S5:** SC-XRD diffraction patterns collected of thick 1T-TaS2 crystals upon rapid cooling at (a) room temperature (RT), (b) 160 K. The presence of continuous Scherrer rings indicates that the thick crystals exhibit polycrystalline characteristics.

**Figure S6:** Temperature-dependent lattice parameters and unit-cell volume of thin 1T-TaS₂ crystals. Lattice parameters a and c (left y-axis) and the corresponding unit-cell volume (right y-axis) were extracted from single-crystal XRD measurements as a function of temperature.

**
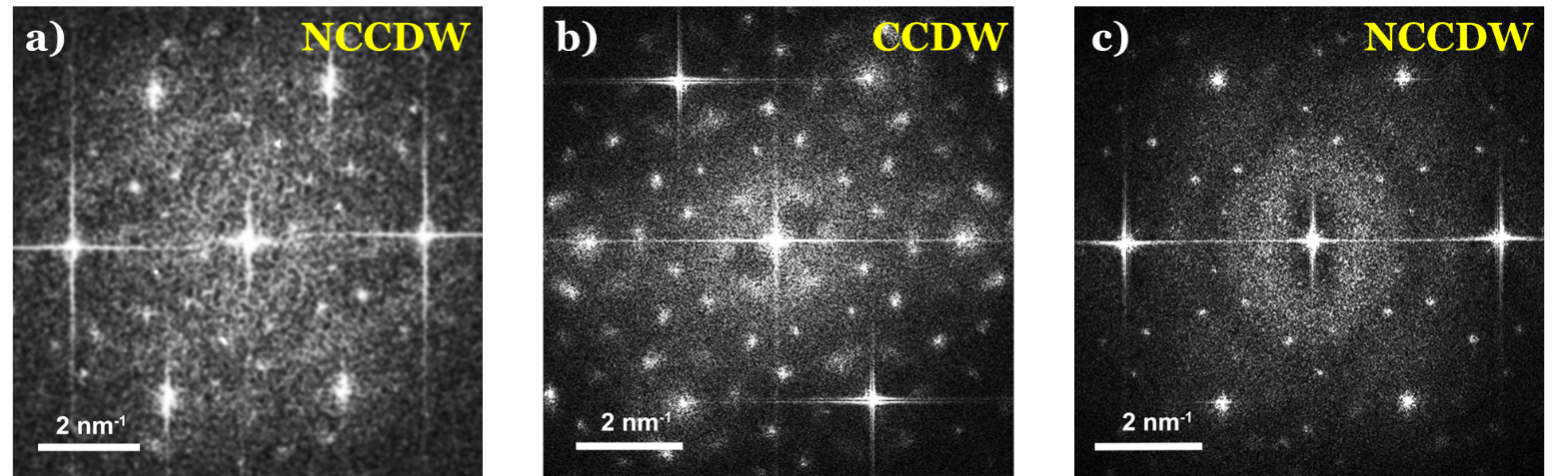
**

**Figure S7:** Fast Fourier transform (FFT) patterns of 1T-TaS₂ nanocrystals corresponding to the HR-TEM images in Fig. 7a–c of the main text. (a) FFT of the original sample at room temperature in the NCCDW phase, (b) FFT of the fast-cooled (FC) island region showing the CCDW superlattice, and (c) FFT of the FC matrix region in the NCCDW phase. These patterns correspond to the color-annotated FFTs shown in Figs. 7d–f, respectively.
